# Malakoplakia in Association with Adenocarcinoma of Sigmoid Colon; A Case Report

**DOI:** 10.30699/ijp.2019.85430.1810

**Published:** 2019-08-01

**Authors:** Nakisa Niknejad, Fatemeh Nili, Mohammad Shirkhoda

**Affiliations:** 1 Department of Pathology, Besat Hospital, Hamadan University of Medical Sciences, Hamadan, Iran; 2 Department of Pathology, Imam Khomeini Hospital Complex, Tehran University of Medical Sciences, Tehran, Iran; 3 Department of Surgery, Cancer Institute, Imam Khomeini Hospital Complex, Tehran University of Medical Sciences, Tehran, Iran

**Keywords:** Malakoplakia, Colon, Adenocarcinoma

## Abstract

Malakoplakia is a rare granulomatous disease of the genitourinary system. Gastrointestinal tract is the second most common site of involvement. It usually mimics a malignancy but its association with adenocarcinoma has been rarely reported.

A 59-year-old male patient with the history of weight loss and rectal bleeding for two months prior to administration was referred to our hospital. Pre-operative CT scan revealed a large sigmoid colon mass with the extension and invasion to the serosal surface as well as multiple regional metastatic lymph nodes. The patient underwent sigmoidectomy with the primary pathologic diagnosis of adenocarcinoma. Pathologic examination revealed a moderately differentiated adenocarcinoma invading peri-colic adipose tissue and inflammatory reaction compatible with malakoplakia at the invasive borders of the tumor with the extension to the serosal surface.

In the patients with gastrointestinal malakoplakia, the presence of possible adjacent malignancy should be screened. The possibility of over-staging should also be considered for adenocarcinoma cases in association with malakoplakia.

## Introduction

Malakoplakia is a granulomatous disease of infectious origin (1). This rare disease is usually seen in the genitourinary system (1, 2). However, different organs such as gastrointestinal tract, skin, lung and central nervous system have also been reported to be affected (1-4). Malakoplakia generally occurs in immunocompromised patients, though atypical presentation in immunocompetent subjects has also been reported (2, 3). Malakoplakia of the gastrointestinal tract and the other visceral organs has been rarely reported (1, 5). Malakoplakia usually mimics a malignancy but in some instances, it may be accompanied with a malignant tumor (6). In this paper, we report a case of gastrointestinal malakoplakia in association with adenocarcinoma.

## Case Report

**Figure 1:**
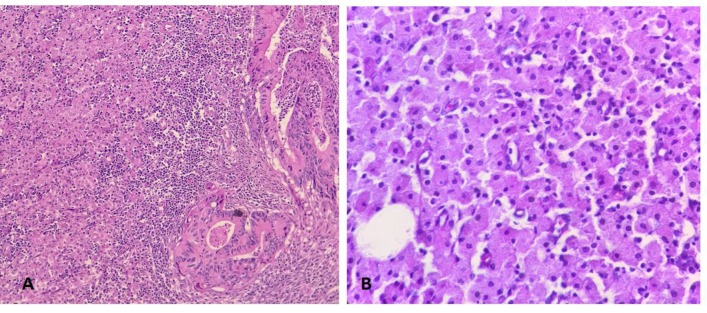
A) H&E stained section (x 100) shows infiltration of inflammatory cells including sheets of histiocytes (left) at the invasive border of adenocarcinoma (right). B) Aggregates of histiocytes with eosinophilic granular cytoplasm (x 400) are shown

**Figure 2:**
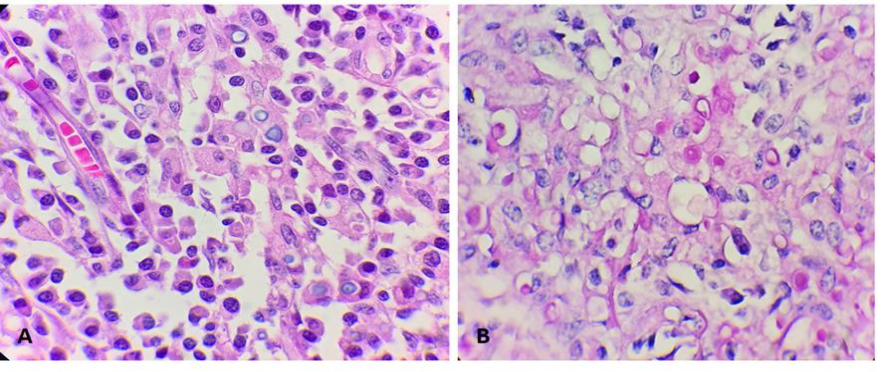
A) Cytoplasmic basophilic inclusions on H&E section are seen, B) Cytoplasmic inclusions were positive on PAS stain (Michaelis-Gutman bodies) (x 1000)

A 59-year-old male patient with the history of rectal bleeding, weight reduction (10 kg within two months) and altered bowel habitus was referred to our hospital. The past medical, drug, and family histories were negative. On the physical examination, the abdomen was normal without any organomegaly. The laboratory study revealed anemia and high serum carcinoembryonic antigen (CEA) of 786 ng/ml. The liver and renal function tests were normal. On colonoscopy, a large mass with the size of 22 to 29 cm was identified from anal verge along with two small polyps located at 10 and 15 cm from anal verge. The computed tomography (CT) scan showed a sigmoid mass with the extension to the serosal surface and involvement of multiple regional lymph nodes without distant metastasis. The pathologic diagnosis was adenocarcinoma, therefore, sigmoid and upper rectal portions were resected with the stapled end to end anastomosis. On the pathologic macroscopic assessment, polypoid sigmoid mass surrounded by seven pedunculated and sessile polyps were identified. Macroscopically, the tumor was extended to the visceral peritoneum, which was suggestive for the pathologic stage T4a. On the microscopic examination, moderately differentiated adenocarcinoma, surrounded by adenomatous and hyperplastic polyps were noted. The tumor invaded into the pericolic adipose tissue through the muscularis propria. Infiltration of the sheets of foamy histiocytes with granular cytoplasm containing concentric basophilic inclusions, which were positive on Periodic Acid Schiff (PAS), Perl’s and Van-kossa stains (Michaelis-Gutman bodies), were observed between and beyond the tumor nests (fig 1,2). This inflammatory process, which extended to the serosal surface and invaded visceral peritoneum, was characteristic of malakoplakia. Four dissected regional lymph nodes were also involved by tumor (pathologic sage pT3N2a)

## Discussion

Malakoplakia is a rare chronic inflammatory process that usually involves genitourinary organs. Gastrointestinal tract is the second most common site of involvement with predominant involvement of colon and rectum (7). Malakoplakia generally affects adult patients with male predominance and is characterized by the accumulation of histiocytes with Michaelis-Gutmann bodies on the pathologic examination (8). 

The suggested pathophysiology of malakoplakia includes unusual inflammatory response to the altered normal flora in association with colon cancer, abnormal immune response and defect in the macrophage-lysosomal function. Infection with some bacteria including *E.coli* and unusual stromal response to the carcinoma are the other possible hypotheses. 

Although malakoplakia usually mimics malignancy, less than 30 cases of malakoplakia in association with adenocarcinoma of colon and rectum have been reported in English literature so far (9-12). Previous reports noted the possibility of over-estimating the pre-operative clinical stage of the disease, which can result in unsuitable treatment of the patients (9). Our patient was a 59 year-old man with pre-operative diagnosis of sigmoid adenocarcinoma and clinical stage of T4aN2 who underwent surgery. On the gross pathologic examination, serosal surface involvement was suggested but tumor involvement on microscopic assessment was up to peri-colic adipose tissue (pT3). Malakoplakia at the invasive border of the tumor was extended to the serosa and led to the over-staging in radiologic and gross examination. 

Jadhav et al (8) reported colorectal malakoplakia in a child with multiple polyps. In our patient, adenomatous and hyperplastic polyps were observed around the main lesion. Asiyanbola et al. presented the oldest case of malakoplakia in a 90-year-old female patient with adenocarcinoma with Dukes' stage B which occurred in association with malakoplakia. They stated that Duck’s stage B is the most common stage of adenocarcinomas associated with malakoplakia in the previous reports (10). Pillary et al (11) reported four cases of malakoplakia associated with the colorectal cancer and reported a tendency for the rectal region involvement in the elderly male subjects. They showed that malakoplakia is usually found at the infiltrating border of the tumor without admixture with the neoplastic glands (11). Karasavvidou et al (12) reported a 64-year-old male patient with cachexia and radiological evidence of metastatic tumor of the liver. In colonoscopy a large malignant polypoid mass was identified in the colon along with multiple distinct polyps throughout the rest of the colon, which was in line with the findings of our reported case. However, our presented patient had no metastasis.

Despite the possibility for errors in clinical or intra-operative over-staging of colorectal adenocarcinoma in association with malakoplakia, the treatment planning and prognosis would not be altered.

## Conclusion

In the patients with gastrointestinal malakoplakia, the presence of possible adjacent malignancy should be screened. The possibility of pre-surgical over-staging for adenocarcinoma in association with malakoplakia must be considered as well.
